# Visual search errors are persistent in a laboratory analog of the incidental finding problem

**DOI:** 10.1186/s41235-020-00235-4

**Published:** 2020-07-29

**Authors:** Makaela S. Nartker, Abla Alaoui-Soce, Jeremy M. Wolfe

**Affiliations:** 1 Department of Psychological and Brain Sciences, Johns Hopkins University, Baltimore, MD USA; 2 Department of Psychology, Princeton University, Princeton, NJ USA; 3 Visual Attention Laboratory, Department of Surgery, Brigham and Women’s Hospital, Boston, MA USA; 4 Department of Ophthalmology and Radiology, Harvard Medical School, Boston, MA USA

**Keywords:** Visual search, Hybrid search, Radiology, Incidental findings, Errors

## Abstract

When radiologists search for a specific target (e.g., lung cancer), they are also asked to report any other clinically significant “incidental findings” (e.g., pneumonia). These incidental findings are missed at an undesirably high rate. In an effort to understand and reduce these errors, Wolfe et al. (*Cognitive Research: Principles and Implications* 2:35, 2017) developed “mixed hybrid search” as a model system for incidental findings. In this task, non-expert observers memorize six targets: half of these targets are specific images (analogous to the suspected diagnosis in the clinical task). The other half are broader, categorically defined targets, like “animals” or “cars” (analogous to the less well-specified incidental findings). In subsequent search through displays for any instances of any of the targets, observers miss about one third of the categorical targets, mimicking the incidental finding problem. In the present paper, we attempted to reduce the number of errors in the mixed hybrid search task with the goal of finding methods that could be deployed in a clinical setting. In Experiments 1a and 1b, we reminded observers about the categorical targets by inserting non-search trials in which categorical targets were clearly marked. In Experiment 2, observers responded twice on each trial: once to confirm the presence or absence of the specific targets, and once to confirm the presence or absence of the categorical targets. In Experiment 3, observers were required to confirm the presence or absence of every target on every trial using a checklist procedure. Only Experiment 3 produced a marked decline in categorical target errors, but at the cost of a substantial increase in response time.

## Significance

Incidental findings are a common source of error in clinical radiology. When a clinically significant finding is missed, the consequences for patients and physicians can be severe. There would be value in reducing these errors but, unfortunately, it is hard to perform extensive tests of potential remedies on clinicians. We have developed an analog of the incidental finding problem for study in the laboratory with non-expert observers. In this mixed hybrid search task, observers look for specific targets (e.g., a picture of a particular flower) and categorical targets (e.g., pictures of *any animal*). Like incidental findings, the categorical targets are missed at a high rate, especially when they are relatively uncommon. In the experiments reported here, we tested three strategies intended to reduce those categorical errors. One of these strategies was successful, albeit with a cost in time, demonstrating that these errors are indeed tractable, at least in this task. These findings get us closer to a solution that could be tried in a clinical setting.

## Background

If you are approaching an intersection in your vehicle, the primary focus of your visual search should probably be the stoplight. But you should also be vigilant for other potential targets, such as pedestrians in the crosswalk, or signs to guide you to your destination. Medical image interpretation presents a similar problem. A radiologist’s search is typically guided by a suspected diagnosis, and this is the primary target of their search. In addition, they are also responsible for reporting any other abnormalities present in an image. A radiologist searching the lung computed tomography (CT) of a patient whose suspected diagnosis is pneumonia would also be responsible for reporting fractured ribs, or nodules that might indicate cancer. Image features that might be clinically significant, but that are not the primary target of the visual search, are referred to in the medical literature as “incidental findings” (Beigelman-Aubry, Hill, & Grenier, 2007). The rate at which incidental findings occur depends on many factors, including the imaging modality, the primary task, and expertise. One review estimated that at least one incidental finding appeared in 24% of a mixed collection of radiologic cases (Lumbreras, Donat, & Hernández-Aguado, 2010; see also Mortani Barbosa & Osuntokun, 2019). An unambiguous estimate of the rate of incidental findings would require an unambiguous definition of what should count as such a finding. Many incidental findings are not clinically significant, and do not necessitate follow-up. Other incidental findings are quite serious and when missed can have grave consequences for patients and physicians. There is significant debate about whether searching for incidental findings is a good idea. Images are often ambiguous. There are often findings that hint at a problem that can only be confirmed or rejected with further testing. That testing comes at a financial and, possibly, a physical cost. Is the additional information gained worth the costs (Oren, Kebebew, & Ioannidis, 2019)? For present purposes, our goal is to help searchers to find targets that they might otherwise miss. One cannot deal appropriately with a finding if one does not find it. We leave the rules for management of these findings to others.

There is a considerable body of literature aimed at quantifying and categorizing errors in radiology. Much less work has been done to investigate the cognitive mechanisms that give rise to those errors. This is due in part to the practical limits on experiments with radiologists as observers and medical images as stimuli. Fortunately, from the vantage point of visual cognition, radiologists can be considered to be just one class of expert human observers. This raises the possibility that we might abstract the crucial elements from the incidental finding problem, study them in the laboratory, and, perhaps, use what we learn to test tractable interventions with radiologists in clinical settings. That is the aim of the work presented here.

**Fig. 1:**
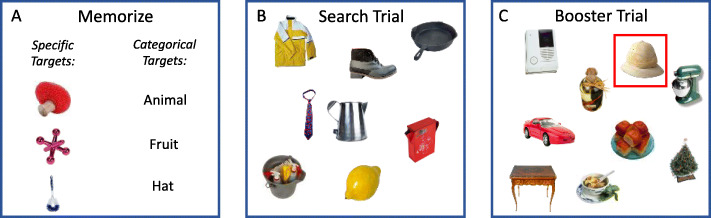
The mixed hybrid search paradigm. **a**. Observers first memorize a set of specific and categorical targets. **b**. After passing a memory test (not pictured), they search for instances of any of these targets in arrays of distractor objects. **c**. In Block 2 of Experiments 1a and 1b, 10% of trials were non-search trials on which one item was highlighted with a red box. Two thirds of the highlighted items were categorical targets, and one third were distractors

In 2017, Wolfe et al. introduced the “mixed hybrid search” task as an analog of the incidental finding problem that could be studied in the laboratory (see Fig. 1). In a “hybrid search” task, observers search visual displays for any of *N* targets held in memory (Schneider & Shiffrin, 1977; Wolfe, 2012a). In mixed hybrid search, non-expert observers memorize a mixture of two types of targets – specific and categorical – and search for any instance of any of those targets. Specific targets are photographs of real objects (e.g., “this mug” or “this car”) and are a stand-in for the primary goal of the radiologist’s search. Categorical targets are names of object categories (e.g., “plants” or “furniture”) and are analogous to incidental findings that could appear in a case. Note that we are not trying to exactly mimic the clinical situation. Looking for a picture of a specific car in a specific pose is not the same as looking for lung nodules, a specific target that can take on a range of appearances. Nor is looking for instances of a category like “animal” exactly like looking for incidental findings. The mixed hybrid search task is like the clinical task in that the searchers are looking for some targets that are more narrowly defined (specific) and some that are less narrowly defined (categorical). Wolfe, Alaoui Soce, and Schill (2017) showed that when categorical targets are rare (only appearing on 10% of trials), observers miss ten times more categorical targets (36.6%) than specific targets (3.6%). Even when categorical and specific targets are equally probable, observers miss 23% of categorical targets against just 9% of specific targets. Like incidental findings in radiology, categorical targets in the mixed hybrid search task produce elevated error rates for a class of targets that observers know they need to report.

Other tasks have also been explored for insights into the incidental finding problem. The “inattentional blindness” phenomenon (Mack & Rock, 1998), for example, involves observers missing targets that they were not looking for, such as an actor in a gorilla suit (Simons & Chabris, 1999) or a gorilla in a lung CT (Drew, Võ, & Wolfe, 2013). These errors are dramatic, but the observers were not looking for gorillas, and gorillas (or other stimuli) had no business being in the lung or the Harvard Psychology Building. Incidental finding errors are errors where the observer misses a target that they were looking for, at least, in a general way. In mixed hybrid search, observers know about all of the targets; so, if they miss a categorical target, it is not because we, as experimenters, were deceiving them. Satisfaction of search (SOS) errors (Berbaum et al., 1990; Tuddenham, 1962), or subsequent search misses (SSM; Cain, Adamo, & Mitroff, 2013) may also be related to the incidental finding problem. These errors arise when the presence of one target makes the detection of a second target less likely. One critical difference between SOS errors and incidental finding errors, however, is that an incidental finding can be the only target present and can be missed, nonetheless. The mixed hybrid search paradigm overcomes these limitations of inattentional blindness and SOS as models of the incidental finding problem.

The experiments presented here tested whether the mixed hybrid search task could be modified to reduce the miss error rate for categorical targets, our stand-in for incidental findings. There is reason to suspect that these errors might be rather stubborn. Wolfe et al. (2007) sought to reduce misses in low prevalence search by targeting subjects’ criterion, but this strategy had limited success. However, some other attempts using cueing have been successful in reducing errors in “T” and “L” (Russell & Kunar, 2012) and mammogram search tasks (Kunar et al., 2017). A strategy that successfully reduces error rates in the mixed hybrid task could provide valuable information about the cognitive mechanisms underlying these errors and could serve as a reasonable starting point for testing in the clinic with experts.

All of the strategies we tested were designed to be potentially transferable to clinical practice. We tested three different strategies in a series of four experiments. The first strategy involved reminding observers about categorical targets by inserting non-search trials where a categorical target was clearly marked. The second strategy attempted to encourage separate searches for specific and categorical targets by requiring observers to respond first to the presence or absence of any specific targets, and then to the presence or absence of any categorical targets. The third strategy involved a version of a checklist procedure, in which observers had to confirm the presence or absence of every target on every trial. Categorical errors proved to be persistent. Only the third strategy, the checklist procedure, was successful in increasing the number of categorical targets detected. These results provide us with information about what does not work and clues to what might work to increase the detection of incidental findings.

## Experiment 1a: “Boosting” categorical targets in memory – three specific, three categorical

### Methods

#### Participants

Twelve observers were included in the analysis (nine women, average age 27 years), consistent with the sample size of Wolfe et al. (2017). Two additional observers did not complete the study and were replaced. All observers had 20/25 vision or better with correction and passed the Ishihara Color Test (Ishihara, 1987). All observers gave informed consent before participating and were paid at a rate of US$11/h. Informed consent procedures were approved by the Partners Human Research Committee, protocol 2007P00646/BWH.

#### Apparatus

All of the experiments reported here were run on an iMac model A1225 (EMC 2211) with a 24″ screen, OSX Version 10.11.6. The screen resolution was 1920 × 1200 pixels with a refresh rate of 60 Hz. The experiment was run using MATLAB 9.0.0 (R2016a) and Psychtoolbox version 3.0.11. (Kleiner, Brainard, & Pelli, 2007). Participants were seated with their eyes approximately 60 cm away from the monitor.

#### Stimuli

The stimuli for all experiments were 750 photorealistic images of isolated objects (Brady, Konkle, Alvarez, & Oliva, 2008) divided into 15 distinct categories: animals, cars, hats, masks, shoes, fruit, furniture, kitchenware, musical instruments, plants, rocks and minerals, signs, sweets, timepieces, and weapons. Each category contained 50 exemplars. Stimuli were presented in random cells of a 5 × 5 invisible grid subtending 960 × 960 pixels on a white background. An individual stimulus subtended approximately 173 × 173 pixels. At the ~ 60-cm viewing distance, these were 4.5 × 4.5° items in a 24.5 × 24.5° field. Within the grid, items were randomly repositioned by ± 0.5° to disrupt the regularity of the display.

#### Procedure

Wolfe et al. (2017) found that observers performed surprisingly well when there were two categorical targets present on a trial. The discovery of one categorical target appeared to have “primed” the detection of the second categorical target, even if the two were from different categories. Based on this observation, we hypothesized that reminding observers about categorical targets throughout the experiment might result in fewer misses. In Experiment 1a, we provided reminders by inserting occasional non-search trials in which a categorical target was clearly marked within the search array.

For each observer, targets were chosen in the following way: first, three specific targets were selected at random from any category. Next, three categorical targets were selected at random from the categories that remained after choosing the specific targets. All remaining images could serve as distractors in both the memory test and the search task. In other words, if one of the targets was a specific image of a cat, then the category “animals” could not serve as a categorical target, but other animals could appear as distractors. Obviously, if the category “animals” was a target category, any animal image would be a target.

Observers began the experiment by memorizing their set of three specific targets and three categorical targets (Fig. 1a). The three specific target images and three category names were first shown to observers one at a time, in isolation, for 3 s each. Observers were then presented with a series of 12 (2 x memory set size) images and asked to indicate whether each image was part of the memory set. Three of the images presented were the specific targets. Another three were categorical targets, where one of the 50 images in each category was chosen at random. The remaining six images were distractors. Observers made a target/non-target response for each image and were required to pass the test with 100% accuracy before moving on to search displays for any of the six targets, held in memory.

Turning to the search task, if a target was present, there was only one. Observers were not told that this would be the case, since in clinical practice, a physician is never certain how many abnormalities might be present. Targets were present on 50% of trials. Of the trials that contained a target, 20% were categorical and 80% were specific. Visual set size (VSS) was randomized across all trials within a block, with displays containing either four, eight, or 12 images (see Fig. 1b for an example). Observers were instructed to press one key for “present” and another key for “absent” (a two-alternative forced choice response) and were asked to respond as quickly and as accurately as possible. Feedback was provided on practice trials. No feedback was given in the experimental trials, because in a clinical setting immediate feedback is not typically available. For search trials with categorical targets, one of the 50 images in that category was chosen at random. Specific targets were the same each time they appeared. Distractors were chosen at random from the non-target categories, including the categories of the specific items (again, if an observer had a specific target image of a cat, any other animal could be a distractor). This meant that across the search trials in Experiments 1a, 2, and 3 (but not 1b), a total of 153 potential targets could appear.

There were three blocks in the experiment with 30 practice trials and 300 test trials in each block. In Blocks 1 and 3, observers performed the original mixed hybrid search task introduced in Wolfe et al. (2017) and described above. Block 2 was the critical “booster” block, in which non-search (“booster”) trials were inserted every 10 trials. On booster trials, a red box surrounded a single object in the display (see Fig. 1c). Observers’ task was simply to indicate whether this object was a target or distractor. Of the booster trials, 33% were target-absent trials, and the remaining 67% were target-present trials in which the boxed object was a categorical target. As we are primarily concerned with reducing the categorical error rates, there were no booster trials where a specific target was marked. In radiology, something similar could be done by inserting previously read cases into the workflow, with abnormalities clearly marked.

## Results

Trials with reaction times (RTs) less than 200 or greater than 10,000 msec were excluded from analysis. The goal is to remove errors of anticipation and extremely long RTs due to motor errors or major lapses of attention. This removed 0.55% of trials. In this experiment, there were no trials with RTs less than 200 msec. All excluded trials were above the 10,000 msec cutoff.

**Fig. 2:**
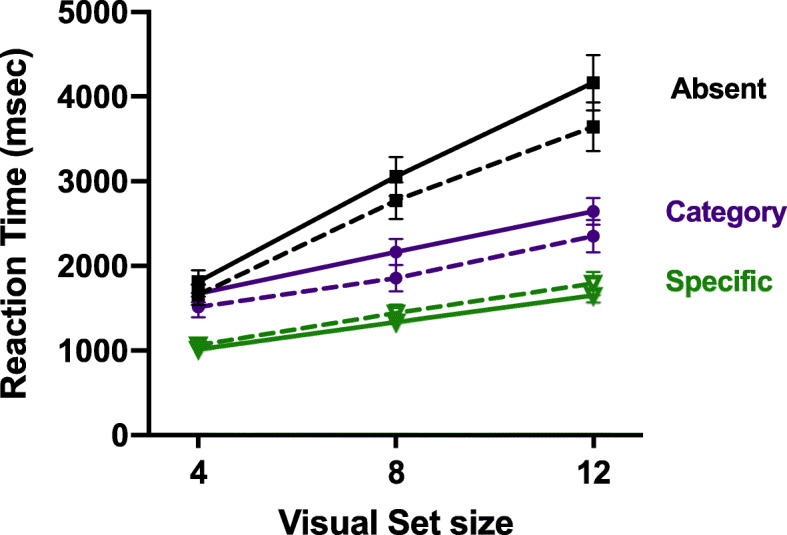
Solid lines show the reaction time (RT) x visual set size functions for the *non-boosted* trials in Experiment 1a, averaged across the 3 blocks. For comparison, dotted lines show data from the 20/80 condition of Experiment 2 in Wolfe et al. (2017). Error bars are ± 1 SEM

Figure 2 shows the RT x VSS functions for all conditions in this experiment, as well as the data from the 20% categorical/80% specific condition from Experiment 2 of Wolfe et al. (2017), for basis of comparison. Data are averaged across observers.

RT patterns replicate the findings of Wolfe et al. (2017), as well as other hybrid search studies (Cunningham & Wolfe, 2014; Cunningham & Wolfe, 2012; Wolfe, 2012a, b). Observers are faster to respond to a specific target than a categorical target, and slowest to respond when no target is present. The search trial RTs from the booster block (Block 2) were similar to the RTs from the no-booster Blocks 1 and 3, so Fig. 2 shows the RT x VSS functions for each condition, averaged across blocks.

**Fig. 3:**
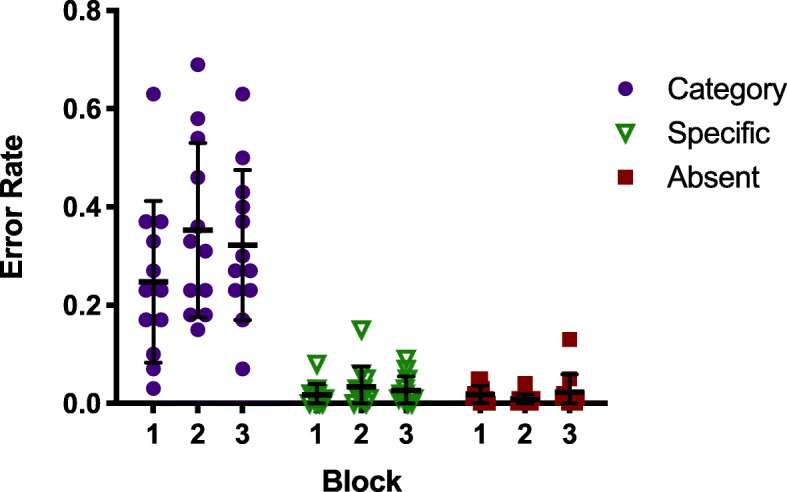
Error rates for all search trials in Experiment 1a, as a proportion of trials of that type. Each dot represents the error rate for one observer. Error bars represent the standard deviation

Our primary interest is in the error rates. We tested the hypothesis that categorical errors would be lower in the booster block (Block 2) than in Blocks 1 or 3. Analyses were performed on the arcsine transformed error rates (Hogg & Craig, 1995). The hypothesis was not supported. Figure 3 shows the error rates for category, specific, and absent trials in all three blocks.

It is clear that errors are not reduced in Block 2. A repeated measures analysis of variance (ANOVA) reveals a strong effect of Condition (Categorical vs Specific *F* (1,11) = 140.431, *p* < .001, *η*^2^_*p*_ = 0.927), but no effect of Block (Booster manipulation *F* (2,22) = 1.949, *p* = .166, *η*^2^_*p*_ = 0.15), and no interaction (*F* (2,22) = 0.88, *p* = .429, *η*^2^_*p*_ = 0.074). These data were also examined with a Bayesian repeated measures ANOVA in JASP. All Bayes Factors (BFs) reported were estimated using the default prior parameters (*r scale fixed effects* = 0.5, *r scale random effects* = 1, *r scale covariates* = 0.354 for ANOVAs; Cauchy scale = .707 for t-tests). The BF_10_ for Condition suggests that these data are 5.558e+16 times more likely under the alternative hypothesis (that categorical and specific error rates differ), than the null. In contrast, the BF_10_ for Block is 0.191, supporting the null hypothesis (that error rates in Blocks 1, 2, and 3 do not differ). On the Block 2 booster trials – the trials with a highlighted item – there were no false alarms (i.e.. responding “present” when a non-target item was highlighted) and only 2% misses (i.e., responding “absent” when a categorical target was highlighted). Observers clearly have no problem recognizing categorical items when search is not required. This is consistent with Russell and Kunar (2012), who found that an exogenous red box cue similar to the one used here reduced miss errors for both high- and low-prevalence targets.

## Discussion

Experiment 1a failed to support the hypothesis that sporadic reminders of the categorical targets would reduce miss errors. This is a null result, raising the possibility that we just did not find the right experimental design. The booster trials were relatively rare (10% of trials) and, since there were three categorical targets, the chance that the next categorical target would be from the boosted category was only one in three. In an effort to give this method more of a chance, we repeated the experiment with a memory set of just one specific and one categorical target.

## Experiment 1b: “Boosting” categorical targets in memory – one specific, one categorical

### Methods

Twelve observers were tested (eight women, average age 24 years). All observers had 20/25 vision or better with correction and passed the Ishihara Color Test (Ishihara, 1987). All observers gave informed consent before participating and were paid at a rate of US$11/h. Informed consent procedures were approved by the Partners Human Research Committee, protocol 2007P00646/BWH.

The methods for this experiment were identical to Experiment 1a, except that the memory set contained just one specific and one categorical target. Thus, there were now just 51 potential targets across the experiment (the single specific image, and any of the 50 exemplars from the target category). Decreasing the memory set size ensured that when a categorical target was “boosted,” another item from that same category was very likely to appear before the next booster trial. We predicted that when observers only had one category to search for, they would benefit more from the priming-like effect of booster trials and detect more of the categorical targets.

## Results

**Fig. 4:**
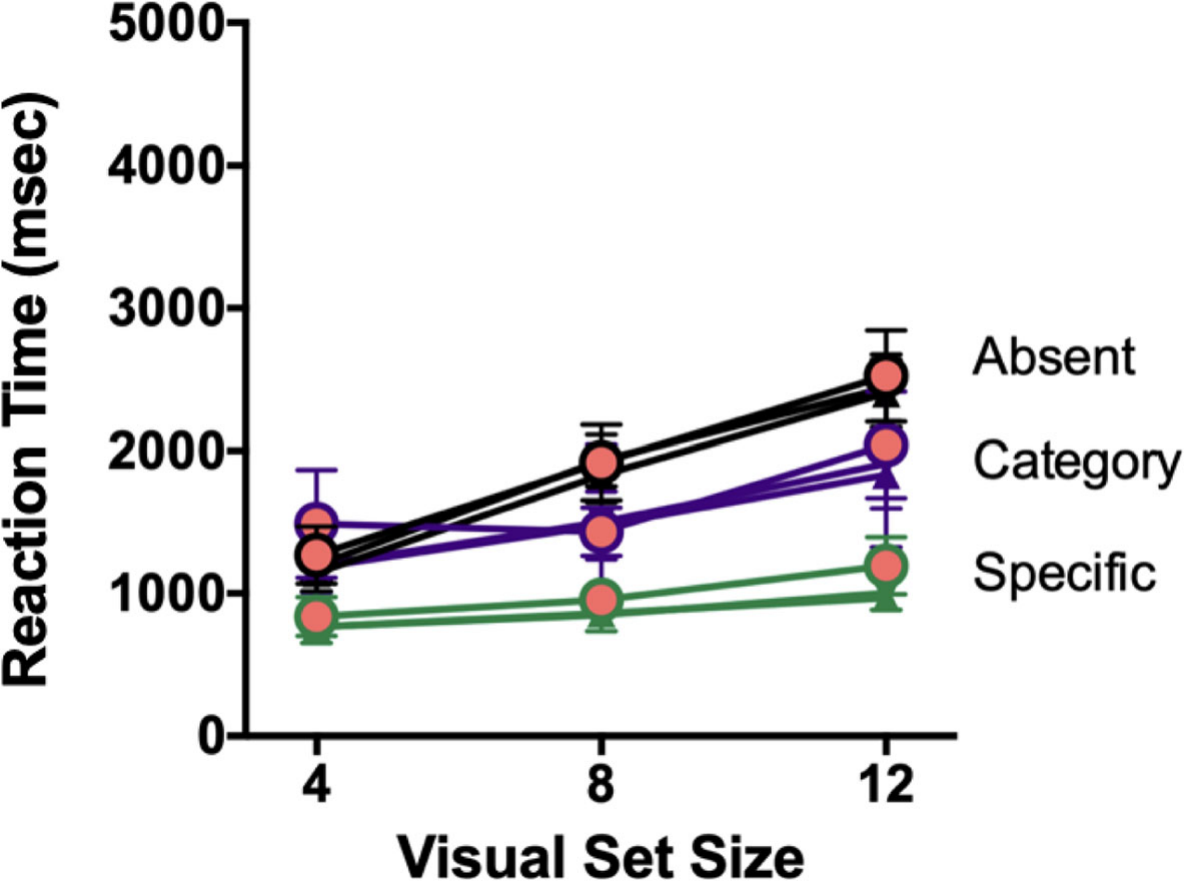
Reaction time (RT) x VSS functions for Experiment 1b for all search trials, averaged across the 3 blocks. Solid symbols are the *non-boosted* trials from Blocks 1 and 3. Large red-filled circles highlight the *non-boosted* trials from Block 2. RTs do not vary systematically with block. Error bars are ± 1 SEM

Trials with RTs shorter than 200 msec or longer than 10,000 msec were excluded from analysis. This removed 0.8% of trials. The pattern of RTs is shown in Fig. 4. Overall, RTs are faster in Experiment 1b than Experiment 1a because the memory set size is smaller (Cunningham & Wolfe, 2014; Wolfe, 2012a, b).

**Fig. 5:**
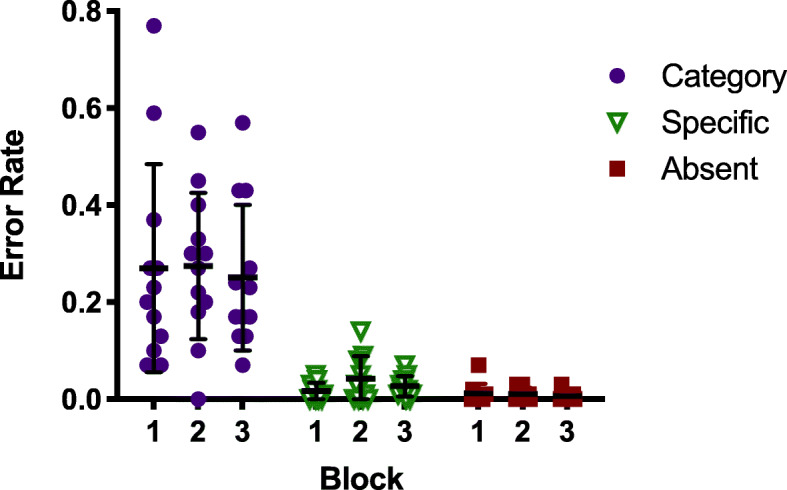
Error rates for all conditions and all Blocks in Experiment 1b, as a proportion of trials of that type. Error bars represent the standard deviation

As before, we are most interested in the error rates. Figure 5 shows the error rates for each search trial type in each block of Experiment 1b.

Again, adding booster trials (Block 2) failed to reduce error rates for categorical targets. There is a main effect of Condition (*F* (1,11) = 70.893, *p* < .001, *η*^2^_*p*_ = 0.866, *BF*_10_ = 1.285e+15), but no main effect of Block (*F* (2,22) = 0.756, *p* = .481, *η*^2^_*p*_ = 0.064, *BF*_10_ = 0.137), and no interaction between Condition and Block (*F* (2,22) = 0.322, *p* = .728, *η*^2^_*p*_ = 0.028, *BF*_10_ = 5.284e+13). Reducing the memory set size to two items did not have a dramatic impact on the miss rate either. Observers still missed 26% of categorical targets in Experiment 1b, compared with 30% in Experiment 1a, a striking indication that the error rates, as seen in Experiment 1a, were not the by-product of a relatively high memory load. Observers continue to miss our analog of incidental findings, even when there is only one category of incidental items to hold in mind.

## Discussion

Reminding observers of categorical targets every ten trials does not improve their detection of other categorical targets in this task. Error rates remain high. There are useful, if negative pieces of information in these data. The results of Experiments 1a and 1b suggest that the “freshness” of categorical targets in memory is not the issue in this task. Observers have not forgotten their categorical targets; they just fail to detect them when they have to search. In addition, searching for even just one of these ill-defined categorical targets is difficult, as demonstrated by the still-high 26% miss rate in Experiment 1b. In Experiments 2 and 3, we move away from attempts at priming categorical targets in memory, toward response-based efforts to reduce errors.

## Experiment 2: Separating the responses for specific and categorical targets

It seems reasonable to conceive of the mixed hybrid task as a task involving two questions: “Are any of your specific targets present here?” and, “Are any of your categorical targets present here?” This would be akin to a radiologist asking, “Does this lung X-ray indicate that the patient has pneumonia?” and, “Is there anything else of note in this image?” In Experiment 2, we test the hypothesis that encouraging a separate search for categorical items would be helpful, by requiring observers to make one response for specific targets and a second response for categorical targets.

## Methods

This experiment was pre-registered on Open Science Framework (osf.io/6aezw). Fourteen observers were tested (nine women, average age 30 years), but only 12 were included in the final analysis. One observer did not complete the experiment due to time constraints. Another had false-positive rates more than 3 standard deviations above the mean. Two additional observers were run to replace these observers. All observers had 20/25 vision or better with correction and passed the Ishihara Color Test (Ishihara, 1987). All observers gave informed consent before participating and were paid at a rate of US$11/h. Informed consent procedures were approved by the Partners Human Research Committee, protocol 2007P00646/BWH.

For Experiment 2, the memory set was three specific and three categorical targets as in Experiment 1a (thus, 153 potential targets could appear across the experiment). The procedure for choosing targets was the same, and observers committed the targets to memory and passed the memory test before moving on to the search trials. Again, one target was present on 50% of trials, and of those targets, 80% were specific and 20% were categorical. Observers completed one block with 30 practice trials and 300 test trials of the mixed hybrid search task. The procedure for the search phase was as follows: a search display was presented, with the question, “Are any specific targets present?” above the display, prompting observers to first indicate the presence or absence of any specific targets. Then, with the same display still visible, the question changed to, “Are any categorical targets present?” and observers then indicated the presence or absence of any categorical targets. Thus, observers made two-alternative forced choice (2AFC) responses on each trial (using the same present/absent keys for each response). The specific prompt always came first to avoid confusion that might have resulted if we randomized the order. We asked the specific question first because specific targets are typically found more quickly. If we asked about categorical first, observers would be frequently withholding a response to the more common specific targets, while waiting for the second question. If observers found a specific target, we could have omitted the second question, since, as in Experiments 1a and 1b, only one target was ever present on a trial. However, observers were not told that this was the case and we did not want to signal this fact via our methods. As the results will show, observers behaved as though they considered that a second target might be present.

## Results

**Fig. 6:**
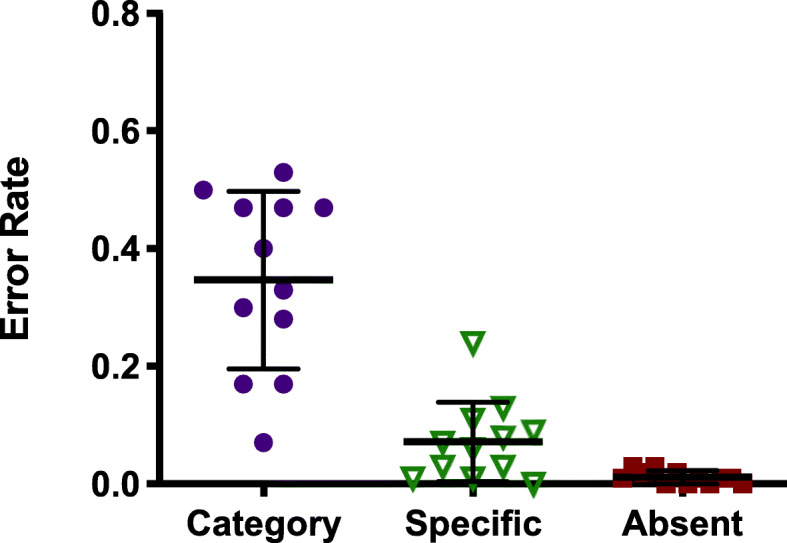
Error rates in all conditions of Experiment 2. Categorical targets are still missed much more than specific targets. Specific errors may have increased slightly, but false alarms are still low

Once again, this strategy fails to reduce errors in the task. Observers missed an average of 35% of categorical targets in this experiment (see Fig. 6). This is comparable to the categorical error rate in prior mixed hybrid experiments (unpaired *t* test on arcsine transformed data for Experiment 2 vs Experiment 1a *t* (22) = 0.74, *p* = .47, Hedges’ *g*_s_ = 0.29). This error rate is far higher, however, than the false-negative rate of 7% for specific targets (*t* (11) = 7.688, *p* < .001, Hedges’ *g*_s_ = 0.82). Due to a bug in the code, all observers were given the same three categories as targets: animals, cars, and hats. Paired *t* tests on the miss rates for each category were all non-significant. Thus, no one category seems to be producing elevated errors. A retrospective examination of the distribution of errors for different categories on all previous mixed hybrid experiments shows that errors are relatively uniform across categories, so it seems unlikely that the use of the same categories for all observers would be a problem here.

**Fig. 7:**
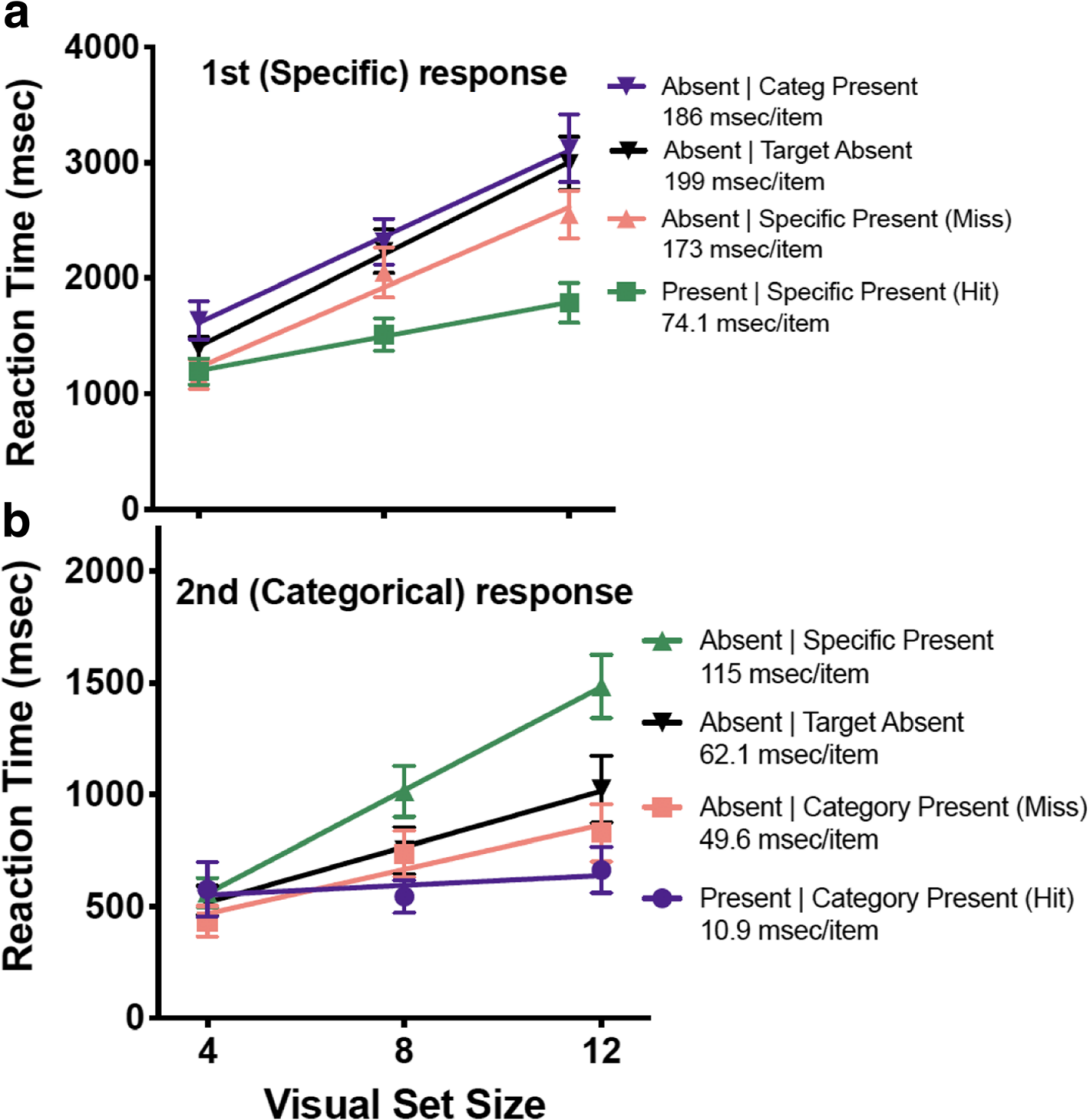
**a** (top). Reaction times as a function of visual set size for the 1st (specific) response. **b** (bottom). Reaction times as a function of visual set size for the 2nd (categorical) response. Each line corresponds to a type of trial. There are two possible true-negative responses for response 1 and response 2: a trial containing no targets at all, and a trial containing a target of the other type

RTs from this experiment give us some insight into why the categorical error rate remains so high. There are two reaction time measurements in this experiment: the time from display onset until the specific prompt response, and the time of the specific prompt response to the categorical prompt response. Figures 7a and b show RTs for each of the responses, with separate lines for each type of response (present/absent) contingent on the type of trial (Specific Present, Categorical Target, and Absent).

Looking first at Fig. 7a, we see standard search results. The slopes for hit trials (a specific response when a specific target is present) are 74 msec/item. Slopes for the three different varieties of absent responses are a bit more than twice that steep (Categorical Present (True negative) = 186 msec/item, Absent (True negative) = 199 msec/item, Specific Present (Miss) = 173 msec/item). These are “inefficient” searches because there are multiple target types in this hybrid search (Wolfe, 2012a). The miss trials (an absent response when a specific target is present) are somewhat faster than the absent trials. This is also a standard result in search tasks; observers miss targets when they respond a bit too quickly (Wolfe, 2012b).

Turning to Fig. 7b, the pattern is somewhat different. All of the RTs are much faster than the first responses (notice that the *y*-axis is half that of 7a). The hit trials (a present response when a categorical target is present) are fast and quite efficient (slope = 11 msec/item). This strongly suggests that observers found the categorical targets while searching for a specific target. They simply had to respond when the categorical prompt appeared. If they had not found a categorical target, they did search for it, but in a relatively cursory manner. The slopes of the absent responses in 7b are between about 50 to 115 msec/item, while the slopes of the absent responses in 7a, given above, are between about 173 to 199 msec/item. Interestingly, observers seemed to search most diligently for a categorical target on those trials when they had already found a specific target (Specific Present (True negative)). Remember, observers were not told how many targets could be present on a single trial. Thus, on trials when the observer had found a specific target, they might have started a new search after responding to the first target. On trials where they found nothing specific, they seem to have wrapped up the search for any categorical targets fairly quickly.

## Discussion

Overall, in Experiment 2 observers missed 7% of specific targets and 35% of categorical targets. Not only does asking observers to make two responses on each trial not reduce categorical errors, it might actually increase specific errors (unpaired t-test on arcsine transformed data for Experiment 2 vs Experiment 1a *t* (22) = 2.085, *p* = .049, but this is a post-hoc analysis and not corrected for multiple comparisons). It could be that the added demands of making two responses produced some careless errors. The relatively fast reaction times for the categorical prompt (1 s to respond on average) indicate that observers are doing most of their searching during the specific prompt (a little more than 2 s to respond on average). The additional time devoted to the second response does not seem to have encouraged an effective second search. Given these RT data, it is not surprising that we do not see an improvement in the detection of categorical targets. Observers did not take the second search seriously enough. In a final effort to induce a more comprehensive search for the categorical targets, Experiment 3 required a separate response for every target.

## Experiment 3: Full checklist procedure

### Methods

This experiment was pre-registered on Open Science Framework (osf.io/meahj). Twelve observers were tested (seven women, average age 28 years). One observer was excluded for error rates more than 3 standard deviations above the mean, and so 11 observers were included in the analysis. All observers had 20/25 vision or better with correction and passed the Ishihara Color Test (Ishihara, 1987). All observers gave informed consent before participating and were paid at a rate of US$11/h. Informed consent procedures were approved by the Partners Human Research Committee, protocol 2007P00646/BWH.

As in Experiments 1a and 2, observers memorized a set of three specific and three categorical targets. After passing the memory test, they completed 30 practice and 300 test trials of the original mixed hybrid search task. This time, a six-response checklist procedure was enforced on every trial. All of the targets in the memory set were listed to the right of the visual display. The list was visible at the same time as the search display. Targets were listed in the order that they were presented during the memorization phase, with specific targets on top, and categorical targets below. The order was the same on every trial. Next to each target item were “yes” and “no” boxes. Observers were required to make a present (click “yes”) or absent (click “no”) decision for all items on the checklist before clicking a separate box on the left side of the display to submit their responses. The number of mouse-clicks, boxes clicked, and reaction time were recorded. To ensure that participants needed to engage with the whole checklist, it was possible for more than one target to be present on each trial. The rules governing the distribution of trial types is shown in Table 1. The resulting distribution of trial types is shown in Table 2. This design is a replication of Experiment 3 from Wolfe et al. (2017). At least one target was present on 70% of trials. The presence of one type of target was independent of the presence of others. In all other respects the experiment followed the same procedure as the previous experiments.

**Table 1:** The constraints on trials in Experiment 3

Constraints	Percentage of trials
Absent trials	30%
One-target trials	45%
Two-target trials	25%
Specific targets	80%
Categorical targets	20%

**Table 2:** The distribution of trial types in Experiment 3

Trial type	Percentage of trials
Absent	30%
One specific target	36%
One categorical target	9%
Two specific targets	16%
Two categorical	1%
One of each	8%

It is worth noting that a checklist, if proven to work, could be tested in clinical settings where checklists have been proposed as a solution for multiple classes of medical error (Gawande, 2010).

## Results

Given that this experiment required several mouse-clicks, trials in which the final RT was shorter than 400 or longer than 30,000 msec were excluded from analysis. This removed 1.7% of trials.

**Fig. 8:**
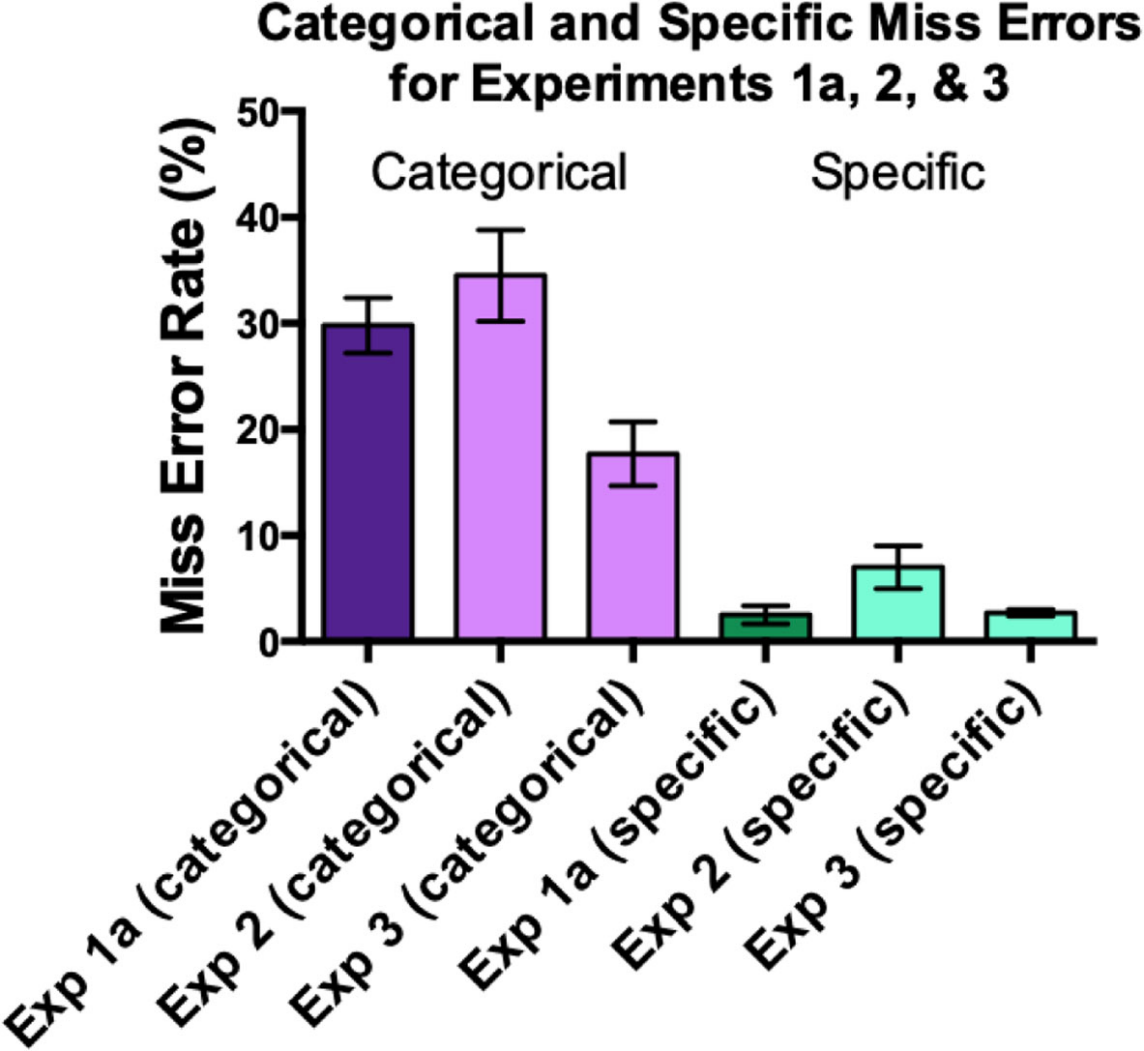
Miss rates for categorical and specific targets in Experiment 3, compared to Experiments 1a and 2. Error bars are ± 1 SEM

The most important data here are the error rates. Miss rates for Experiment 3 are shown in Fig. 8 with average results for Experiments 1a and 2 shown for comparison. While categorical errors remain elevated (~ 18%), they are significantly reduced from the levels in the previous experiments. Unpaired *t* tests, performed on arcsine transformed error rates, show that the categorical miss error rate is significantly lower in Experiment 3 than Experiment 1 (*t* (21) = 3.19, *p* = .004) and Experiment 2 (*t* (21) = 3.03, *p* = .006). The results are essentially unchanged if one looks only at Experiment 3 trials with one target present. The miss error rate for specific targets is not significantly lower in Experiment 3 than in Experiment 1 (*t* (21) = 0.87, *p* = .39) or Experiment 2 (*t* (21) = 1.68, *p* = .10). Within Experiment 3, trials with one specific target can be compared with those with two specific targets. When two specific targets are present, the specific target miss error rate is somewhat elevated, rising from 1.3% to 4.5% (*t* (10) = 4.9, *p* < .001). This could be a “satisfaction of search” effect (Berbaum et al., 1990; Berbaum, Franken, Caldwell, Shartz, & Madsen, 2019; Nodine, Krupinski, Kundel, Toto, & Herman, 1992).

**Fig. 9:**
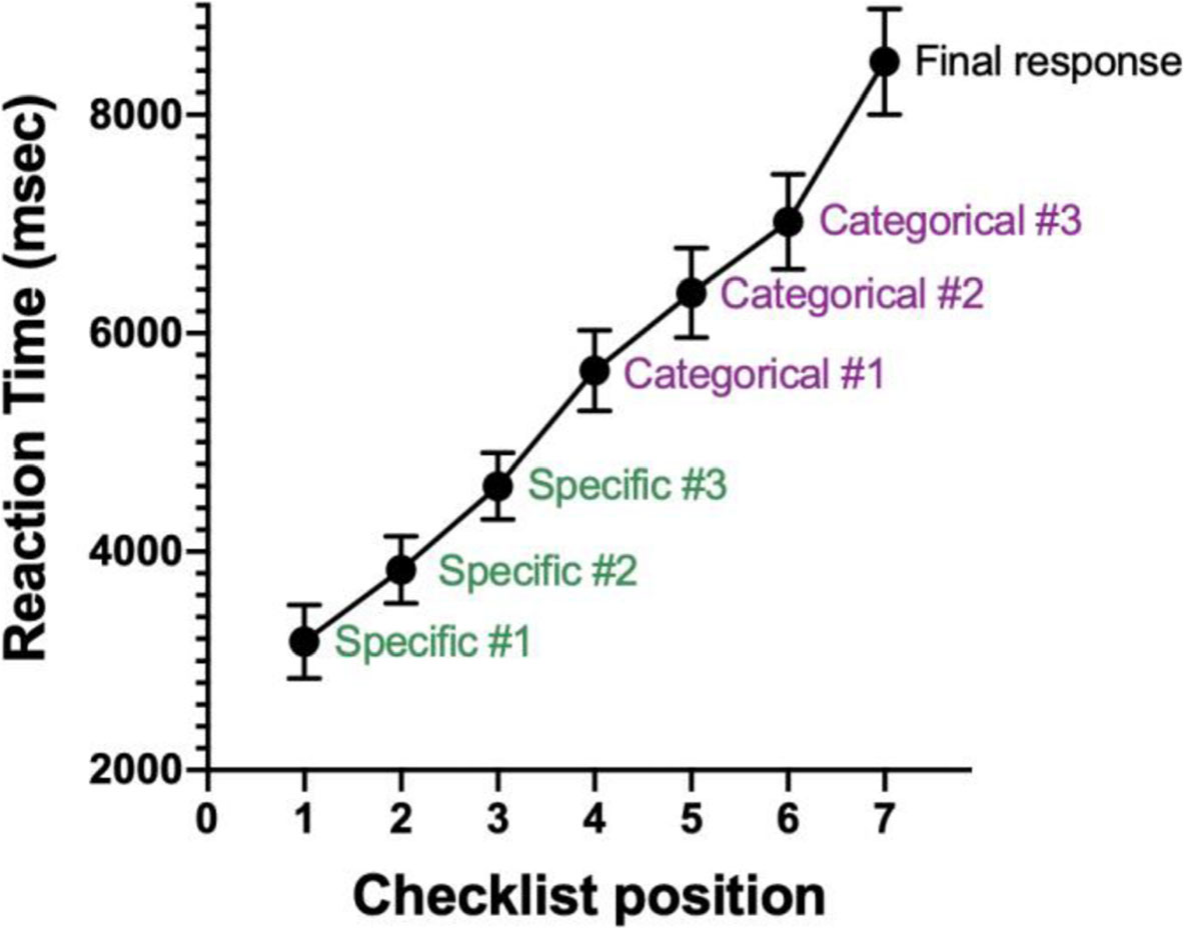
Average reaction times (RTs) for each of the 7 responses in Experiment 3. Error bars are ± 1 SEM

In Experiment 3, observers had to make a minimum of seven clicks on every trial. The average response time for an entire trial was about 8.5 s. Obviously, this is a very significant increase relative to previous experiments. The pattern of RTs for each item on the checklist is relatively uninteresting. Observers tend to start at the top and work their way down. This is shown in Fig. 9. The slope of the roughly linear function is 866 msec/item. RTs for individual clicks on the checklist will be a function of both the type of target(s) present and each target’s position in the list, making the RT x VSS functions for different trial types difficult to interpret. Participants were not told how many targets could appear on a given trial, but they are slowest when one specific and one categorical target are present. In general, participants are slower to respond when at least one categorical target is present.

## Discussion

Unlike the previous experiments reported here, the comprehensive checklist was successful in reducing the miss rate for categorical targets from 30% or more in other experiments to about 18% in this case. This is encouraging but still not ideal. First, a 18% error rate is still high, especially in a task where the targets are unambiguous. When our observers’ attention was directed to single items on “booster” trials in Experiments 1a and 1b, they had no problem deciding whether an item was a member of a category like “fruit” or “furniture,” as is made clear by the 98% accuracy on those trials. Nevertheless, in Experiment 3, 18% of categorical items still did not attract adequate attention during search to permit that identification. Second, the improvement in accuracy was accompanied by a substantial increase in RT over other mixed hybrid search experiments. Given that this experiment required six separate responses, and those responses were made by mouse-click instead of keypress, the increase was to be expected. Still, how we think about the practicality of using full checklists in a clinical setting depends on whether the additional time is seen as additive or multiplicative. If one imagines a case in the clinic taking several minutes to read, adding a few seconds of reporting time might be an acceptable cost for the benefit of reduced errors. On the other hand, if the checklist effect is multiplicative, doubling or tripling the time per case would probably be unacceptable. In fact, the cost is probably in between these extremes. Presumably the checklist does induce more search, so the time cost would not be limited to the cost of a few extra clicks. However, it seems unlikely that radiologists would conduct separate full searches for each item on the list. Follow-up research with a different, more difficult search task would help to document the nature of the cost. For the present, the conclusion from Experiment 3 would be that a full checklist decreases errors but at what is probably a noticeable cost in time per case.

## General discussion

Mixed hybrid search is intended to be a laboratory analog of one of the many challenges facing radiologists and other medical professionals when they evaluate medical images. Medical experts typically have some specific task when faced with an image, but they are also asked to report incidental findings from a less well-defined list of findings that may be clinically significant. In mixed hybrid search, this is modeled by having non-expert observers search for highly specific targets in the form of memorized photographs of objects, as well as less precisely defined, but still well-learned categories of objects. The experiments in this paper replicate the original mixed hybrid findings in Wolfe et al. (2017). Observers miss many more categorical targets than specific targets, especially when the categorical targets are rarer than the specific targets. The results presented here also underline how difficult it is to reduce categorical errors in this task. Experiments 1a and 1b failed to reduce errors by inserting “booster” trials to remind observers about the categorical targets. Experiment 2 found no benefit from enforcing separate responses for specific and categorical targets. Only the full checklist of Experiment 3 produced a significant reduction in categorical errors by requiring separate responses for every target in the memory set. Even so, the error rate dropped to a still high 18%.

Why are observers missing items from target categories? Kundel, Nodine, and Carmody (1978) divided errors on radiological search tasks into three categories: search errors – where the eyes never fixated on the target, recognition errors – where the eyes landed on the target but moved off quickly, and decision errors – where observer scrutinized the target but failed to correctly report it. The ceiling levels of accuracy (98%) on booster trials, when attention was directed to categorical items, suggests that they are able to identify them with very high accuracy when isolated in the display. In other words, the missed categorical targets probably do not represent decision errors. Observer are either not looking at the categorical targets or, perhaps more likely, they are looking at categorical targets but not successfully evaluating their categorical status (a form of recognition error).

Even if one agreed that the mixed hybrid task can serve as a laboratory analog of the incidental finding problem in radiology, the strategies reported in this paper are not going to solve that problem. The checklist approach is promising, but one would want to use a more difficult and/or ambiguous analog of incidental findings in order to determine if the accuracy benefit remained and if the time cost was likely to be acceptable. In a lung or an abdomen, there is a very long list of possible incidental findings. Clearly, clinicians would rebel against a protocol that required a specific response to the presence or absence of each one of these.

Nevertheless, further experiments using this task could get us closer to a solution that could be tried in a clinical setting. For example, anecdotally, some clinicians have told us that they like to look at an image first as a “naïve observer,” with no specific task (“is there *anything* of interest here?”). Then, they look at the medical record to see why, specifically, they are being asked to evaluate this case. That could be tried in our mixed hybrid task if we used a protocol in which observers searched for categorical targets first and, only after giving that response, would they be asked about specific target(s) for the trial. This would require a version of mixed hybrid search with different specific targets on each trial. While that would be a poor analogy to a task like lung cancer screening, where the specific target is the same all day, it might be quite a good model for general radiology, where the clinician is fielding a wide variety of different types of images from case to case.

These experiments add to our understanding of whether satisfaction of search can explain errors in this search task. Recall that part of the motivation for Experiments 1a and 1b was the observation of a reverse SOS effect for categorical targets in Wolfe et al. (2017). Observers were *more likely* to find a second categorical target if they had already found a different target. In Experiment 2 of this paper, we find an analogous effect: search slopes for a categorical target were inefficient when observers had already found a specific target, suggesting that they embarked on a serious search for a categorical target. Unfortunately, we do not know whether they would have detected more categorical targets in this situation, since only one target was ever present in Experiment 2. In Experiment 3, where two targets could occur on the same trial, we do observe a classic SOS effect on trials where two specific targets are present. Error rates rise from 1.3% to 4.5%. When one target is specific and one is categorical, the chance of missing the specific target is intermediate, 2.5%. We are more interested in possible SOS effects for categorical targets. There is an 18.5% miss rate for categorical items that are the only target on a trial. When there is one categorical and one specific target, observers miss essentially the same, 18.1% of targets. Thus, there is no evidence that finding a specific target has any particular impact on finding a categorical target.

Finally, quite apart from any connection to real-world problems in radiology, the mixed hybrid task provides a useful tool to study inattentional blindness and related phenomena (Cohen, Cavanagh, Chun, & Nakayama, 2012; Simons & Levin, 1998). Mixed hybrid search makes it clear that you can miss something that is unambiguously visible even if you know that you are looking for it. Mixed hybrid search is a form of inattentional blindness that does not require an unexpected item and is not limited to a single trial. Observers will miss 20–40% of items across dozens of opportunities to find them. Inattentional blindness is not simply a compelling and entertaining phenomenon. It is a part of routine visual perception and cognition. Under some circumstances, including in clinical radiology, it presents a problem. The experiments in this paper show this problem to be persistent in the face of efforts to “cure” it.

## Data Availability

Experiments 2 and 3 were pre-registered on Open Science Framework, and those original projects can be viewed here: https://osf.io/6aezw/ (Experiment 2), https://osf.io/meahj/ (Experiment 3). All raw data and links to these pre-registrations are available here: https://osf.io/dy9zu/.

## Abbreviations

SOS: Satisfaction of search
SSM: Subsequent search misses
VSS: Visual set size
